# Hydrographic shipboard profile data collected within Olympic coast national marine sanctuary, 2005–2023

**DOI:** 10.1016/j.dib.2024.110171

**Published:** 2024-02-07

**Authors:** Craig M. Risien, Kathryn R. Hough, Jeannette Waddell, Melanie R. Fewings, Brandy T. Cervantes

**Affiliations:** a College of Earth, Ocean, and Atmospheric Sciences, Oregon State University, Corvallis, OR 97331, USA; b National Oceanic and Atmospheric Administration, Olympic Coast National Marine Sanctuary, Port Angeles, WA 98362, USA

**Keywords:** Seawater temperature, Practical salinity, Density, Dissolved oxygen, CTD, Northern california current, Washington state

## Abstract

Olympic Coast National Marine Sanctuary (OCNMS), which was established in 1994 and covers an area of 8257 km^2^, is located along Washington State's remote and rugged outer coast towards the northernmost extent of the California Current System (CCS). In this region, summertime equatorward winds drive seasonal upwelling of cold, nutrient rich waters onto the continental shelf. These waters help fuel a highly diverse and productive ecosystem that includes marine mammal and seabird communities as well as commercially and culturally important fisheries. The sanctuary is located within the boundaries of the legally defined Usual and Accustomed (U&A) fishing grounds of four Coastal Treaty Tribes, the Hoh Tribe, Makah Tribe, Quileute Tribe, and the Quinault Indian Nation, which hold treaty fishing rights and co-manage fisheries and other natural resources within the sanctuary through state, federal, and international partnerships and agreements. This data article describes shipboard hydrographic Conductivity-Temperature-Depth (CTD) and dissolved oxygen profile data that were collected within the sanctuary at fourteen locations during mooring deployment, recovery, and maintenance cruises between the months of May and October from 2005–2023. The 792 CTD profiles were acquired using Sea-Bird Scientific 19 SeaCAT or 19plus SeaCAT CTD profilers with associated SBE-43 (Sea-Bird Electronics) or Beckman or YSI-type (Yellow Springs Instruments) dissolved oxygen sensors. The data were processed using Sea-Bird Scientific's SBE Data Processing application. These data are needed for improving our understanding of subsurface oceanographic conditions — including marine heat waves, changes in timing of spring transition to upwelling, seasonal hypoxia, and ocean acidification — in this important but undersampled region, and can be used to help improve the management of marine resources regionally and within the sanctuary. The CTD cast data are available via Zenodo at https://doi.org/10.5281/zenodo.10466124.

Specifications Table

**Table:** 

Subject	Oceanography
Specific subject area	Hydrographic data collected within Olympic Coast National Marine Sanctuary (47.13–48.51°N, 124.18–125.68°W)
Data format	Raw Filtered
Type of data	Hydrographic Figure
Data collection	This data collection consists of 792 raw and processed Conductivity-Temperature-Depth (CTD) and dissolved oxygen profiles that were collected within Olympic Coast National Marine Sanctuary during mooring deployment, recovery, and maintenance cruises between the months of May and October from 2005–2023. These data were acquired using Sea-Bird Scientific 19 SeaCAT or 19plus SeaCAT CTD profilers with associated SBE-43 or Beckman or YSI-type dissolved oxygen sensors. They were processed using Sea-Bird Scientific's SBE Data Processing (v7.26.7) application.
Data source location	Hydrographic data collected within Olympic Coast National Marine Sanctuary **Station Name Latitude Longitude Water Depth (m, MLLW)** *Makah Bay (MB)* MB01548.3254^o^N124.6768^o^W15 MB04248.3240^o^N124.7354^o^W42 *Cape Alava (CA)* CA01548.1663^o^N124.7568^o^W15 CA04248.1660^o^N124.8234^o^W42 CA06548.1659^o^N124.8949^o^W65 *Teahwhit Head (TH)* TH01547.8761^o^N124.6195^o^W15 TH04247.8762^o^N124.7334^o^W42 TH06547.8767^o^N124.7967^o^W65 *Kalaloch (KL)* KL01547.6008^o^N124.4284^o^W15 KL02747.5946^o^N124.4971^o^W27 KL05047.5933^o^N124.6112^o^W50 *Cape Elizabeth (CE)* CE01547.3568^o^N124.3481^o^W15 CE04247.3531^o^N124.4887^o^W42 CE06547.3528^o^N124.5669^o^W65
Data accessibility	Repository name: Zenodo Data identification number: 10.5281/zenodo.10466124 Direct dataset link: https://doi.org/10.5281/zenodo.10466124 Zenodo is an open repository operated by the European Organization for Nuclear Research, known as CERN.

## 1 Value Of The Data

• The data presented here can be used for model validation analyses [1,2] in this oceanographically and ecologically important but undersampled region.
• This CTD data set can be used as part of the process to quality control associated OCNMS mooring data [3] via cross-sensor calibrations and comparisons, which can correct for sensor drift.
• Using the long time series presented here, researchers can characterize regional ocean change at intra and interannual time scales as well as place extreme events [4] in greater context.

## 2 Background

The nineteen years (2005–2023) of CTD and dissolved oxygen profile data described in this data article were previously not publicly available. Making these data publicly available helps advance the understanding of subsurface oceanographic conditions within Olympic Coast National Marine Sanctuary and can be used to improve the management of commercially and culturally important marine resources. These profile data have been processed using the same procedures as those used to process CTD data collected along the Newport Hydrographic Line [5] located off the central Oregon coast, which allows for regional comparisons.

## 3 Data Description

**Fig. 1:**
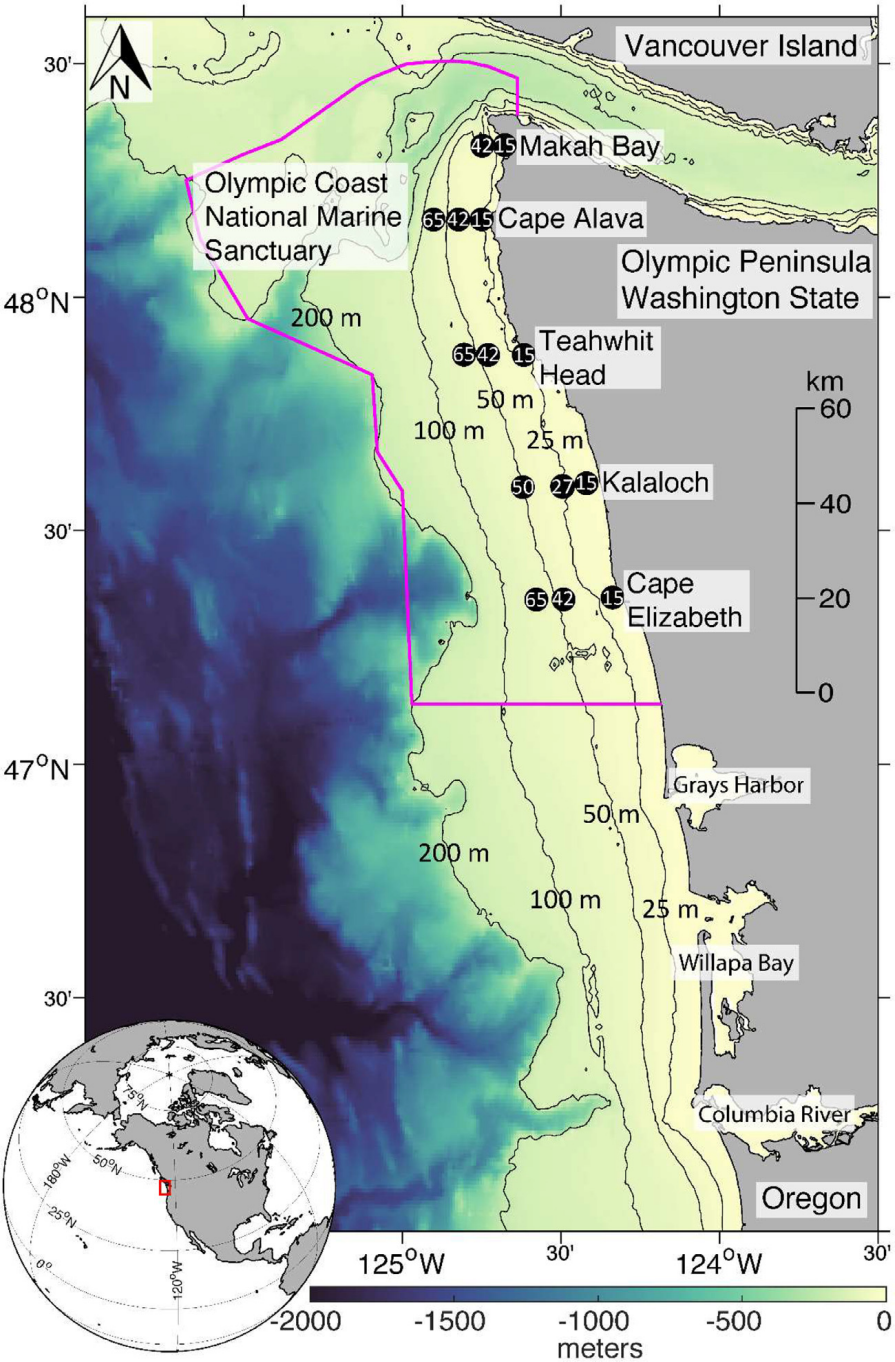
A regional map showing GEBCO bathymetry [7] including the 25, 50, 100, and 200-m isobaths offshore of Washington State's Olympic Peninsula. The boundary of Olympic Coast National Marine Sanctuary is shown in magenta. The 14 mooring sites are shown as black circles with the numbers inside the black circles indicating the water depth at MLLW in meters. The inset map shows North America with the geographic bounds of the regional map indicated as a red box off the Washington coast.

**Fig. 2:**
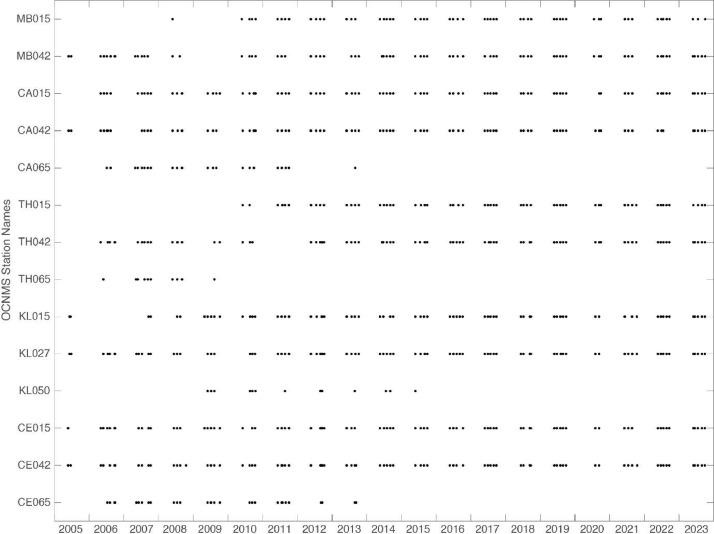
CTD data availability at each of the 14 mooring locations. Each black circle represents an individual CTD cast.

**Fig. 3:**
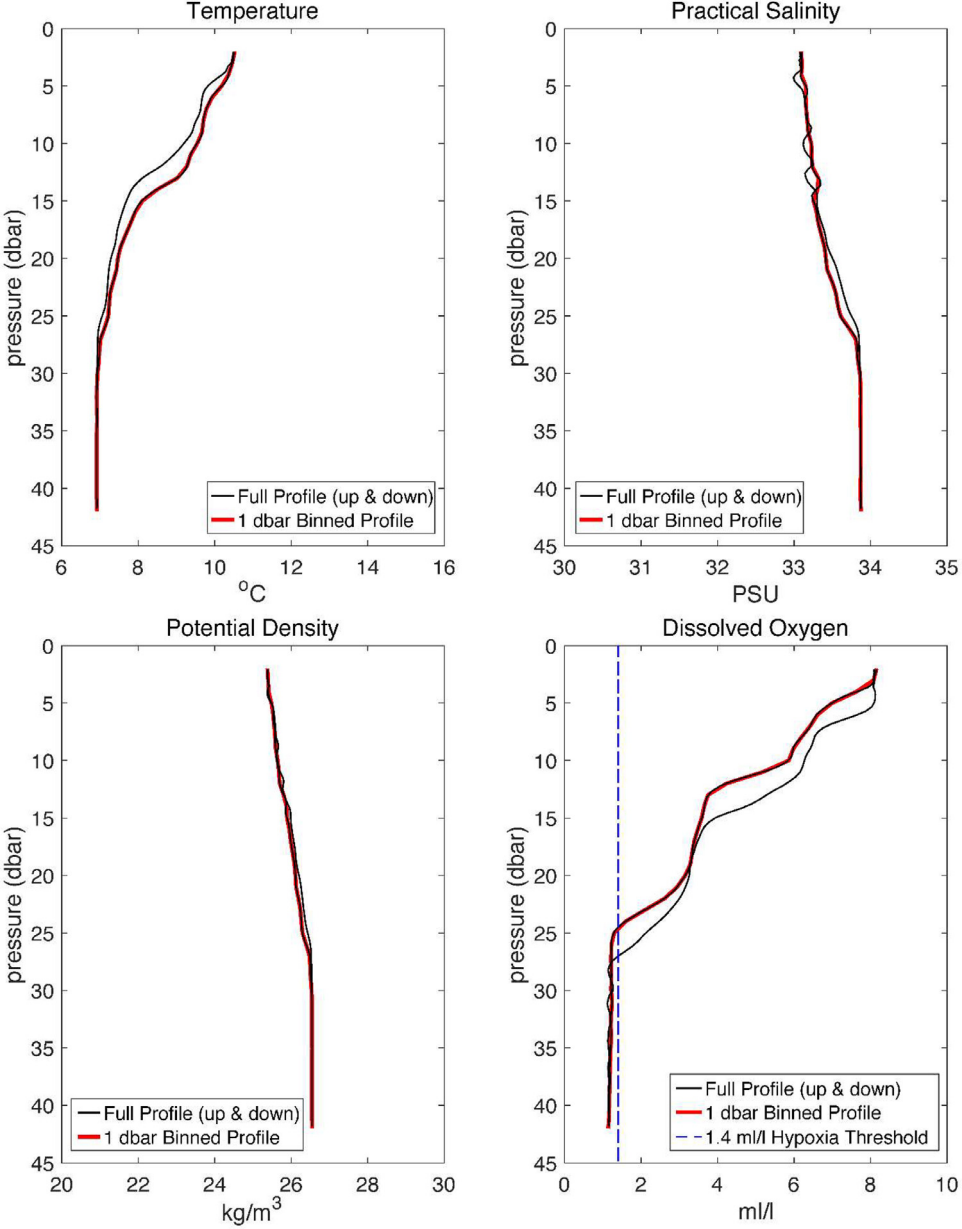
Example temperature (upper left), practical salinity (upper right), potential density (lower left), and dissolved oxygen (lower right) profiles that were recorded at the 42-m Cape Elizabeth site on 25 July 2013. Full profiles (up and down casts) are shown in black. 1 dbar binned downcast profiles are shown in red. The dashed blue line in the lower right panel shows the 1.4 ml/l hypoxia threshold.

The 792 CTD and dissolved oxygen profiles described here are available via Zenodo at https://doi.org/10.5281/zenodo.10466124 [6]. The data set consists of the instrument configuration and calibration files (.con and .xmlcon) derived from annual vendor conducted sensor calibrations and the individual cast data files (.hex and .cnv) that contain CTD and dissolved oxygen observations collected between May 2005 and October 2023 at fourteen hydrographic stations located within Olympic Coast National Marine Sanctuary (Figs. 1 and 2). Additionally, the data set includes 792 CF (Climate and Forecast) compliant NetCDF files (Table 1) that contain the processed (original and 1 dbar vertically bin averaged) temperature, practical salinity, potential density, and dissolved oxygen data for each CTD cast (Fig. 3).

**Table 1:** Names, descriptions, and units of variables included in processed NetCDF files. NetCDF files are named using the convention sitename_yyyymmddThhmmss(_binned).nc with times given in Coordinated Universal Time (UTC). For example, a CTD cast that was conducted at the Cape Elizabeth 42-meter site on 28 August 2013 at 13:10:45 UTC is named CE042_20130828T131045.nc. The associated file that contains 1-dbar bin averaged profiles for this cast is named CE042_20130828T131045_binned.nc.

Variable name	Variable description	Units
Time	CTD cast start time	seconds since 1970-01-01 00:00:00 UTC
Pressure	Seawater pressure	dbar
Depth	Seawater depth calculated from pressure	meters
Temperature	Seawater temperature	°C
Salinity	Seawater practical salinity	1
potential_density	Potential density of seawater calculated from absolute salinity, potential temperature with respect to a reference seawater pressure of 0 dbar	kg m^−3^
dissolved_oxygen	Seawater dissolved oxygen concentration	ml l^−1^
Latitude	Latitude	°N
Longitude^a^	Longitude	°E

a The Specifications Table above lists the longitudes of the CTD casts in °W, but the processed NetCDF file contents use °E to follow the CF standard.

## 4 Experimental Design, Materials and Methods

The 792 CTD+DO profiles were processed using Sea-Bird Scientific's SBE Data Processing (v7.26.7) application, which includes a series of data processing modules [8]. The data presented here were processed using six of the modules in the following order: *Data Conversion, Filter, Align CTD, Loop Edit, Derive, and Bin Average*. The *Cell Thermal Mass* (CTM) module was not applied to the data because the SBE Data Processing manual [8] states that the correction is negligible in regions that do not have steep temperature gradients. In regions that do have steep temperature gradients, the correction is about 0.005 PSU. These processing steps, including not applying the CTM module to the data, are the same as those used to process CTD data that make up the Newport Hydrographic Line time series located off the central Oregon coast [5] thus allowing for a direct comparison between the two regions.

The raw cast data files (.hex), which include the upcast and downcast data (time, pressure, conductivity, temperature, and dissolved oxygen) were converted to ASCII files (.cnv) using the *Data Conversion* module and associated instrument configuration and calibration files (.con and .xmlcon). For the years 2005–2007 when Beckman or YSI-type sensors were used to measure dissolved oxygen, data were converted using the Owens-Millard equation [9]. For the period 2008–2023 when SBE-43 oxygen sensors were used to measure dissolved oxygen, data were converted using the SBE-43 equation, which is a modified version of the Owens-Millard equation [10]. Converted pressure, conductivity and temperature data were then filtered using the *Filter* module. Pressure data collected using SBE 19 SeaCAT or SBE 19plus SeaCAT CTD profilers were filtered using a 2-s or a 1-s low-pass filter, respectively. Conductivity and temperature data were filtered using a 0.5-s low-pass filter. Profiles were then aligned, using the *Align CTD* module, by advancing temperature and conductivity observations by 0.5 and −0.5 s, respectively. Oxygen data were advanced 5 s. Next the *Loop Edit* module, which removes scans associated with pressure slowdowns and reversals, was applied to the aligned data. Scans where the CTD vertical speed was less than 0.25 m s^−1^ were excluded. Additionally, surface (1 m depth) soak data and bad data scans were omitted. After applying the *Loop Edit* module to the data, the *Derive* module was used to calculate depth (meters), practical salinity (PSU), and potential density (kg m^−3^). Finally, all upcast data were excluded and downcast data were binned to 1-dbar bins using the *Bin Average* module. Bad scans were excluded from the binning process. Fig. 3 shows an example CTD cast that was collected at the 42-m Cape Elizabeth site on 25 July 2013. The temperature, practical salinity, potential density, and dissolved oxygen profiles shown in Fig. 3 have been processed using the SBE Data Processing modules and procedures described above.

## Limitations

The processing steps described above did not include correcting for sensor drift within a field season. However, sensors were vendor calibrated annually prior to the start of each field season. Sea-Bird Scientific estimates that the typical drift rates associated with CTD temperature, conductivity and pressure sensors are 0.0002 °C/month, 0.0001 Siemens/meter/month, and 0.0015–0.004% full scale/month, respectively [11]. The calibration drift rate of the SBE-43 is estimated to be less than 0.5% over 1000 h of operation [12].

## CRediT authorship contribution statement

**Craig M. Risien:** Writing – original draft, Data curation, Validation. **Kathryn R. Hough:** Writing – review & editing, Data curation, Validation. **Jeannette Waddell:** Supervision, Writing – review & editing. **Melanie R. Fewings:** Supervision, Writing – review & editing. **Brandy T. Cervantes:** Writing – review & editing.

## Declaration of Competing Interest

The authors declare that they have no known competing financial interests or personal relationships that could have appeared to influence the work reported in this paper.

## Data Availability

Conductivity–Temperature–Depth (CTD) and dissolved oxygen profile data from shipboard surveys collected within Olympic Coast National Marine Sanctuary, 2005–2023 (Original data) (Zenodo).
